# Supplementary material for: Comparison of Conservative Treatment of Cervical Intraepithelial Lesions with Imiquimod with Standard Excisional Technique Using LLETZ: A Randomized Controlled Trial
Source: J Clin Med. 2021 Dec 10;10(24):5777. doi: 10.3390/jcm10245777 (PMC8706260; doi:10.3390/jcm10245777)
Supplement: Supplementary file 1 [file jcm-10-05777-s001.zip › jcm-1506933-supplementary.pdf]

Table S1: Reported side effects.

Side effects		Group 1 (imiquimod)		Group 2 (LLETZ) n = 52
		Before 10 weeks n = 52	After 10 weeks n = 43	
Side effects yes [n (%)]		42 (80.8)	33 (76.7)	23 (44.2)
Grade 1 [n (%)]	Vaginal inflammation	8 (15.3)	8 (18.6)	3 (5.8)
	Vaginal discharge	2 (5.8)	0 (0)	0 (0)
	Vaginal pain	5 (9.6)	0 (0)	0 (0)
	Vaginal haemorrhage	8 (15.3)	6 (14.0)	0 (0)
	Abdominal or lower back pain	8 (15.3)	5 (11.6)	4 (7.7)
	Headache	1 (1.9)	2 (4.7)	0 (0)
	Malaise	6 (11.5)	8 (18.6)	7 (13.5)
	Fever	1 (1.9)	4 (9.3)	0 (0)
	Vertigo	2 (5.8)	2 (4.7)	0 (0)
	Vaginal dryness	4 (7.7)	3 (7.0)	4 (7.7)
	Vaginal stricture	3 (5.8)	4 (9.3)	5 (9.6)
	LUTS	6 (11.5)	10 (23.3)	2 (5.8)
	Nausea	2 (5.8)	2 (4.7)	0 (0)
	Flu-like symptoms	6 (11.5)	4 (9.3)	0 (0)
	Anxiety	5 (9.6)	4 (9.3)	2 (5.8)
	Insomnia	1 (1.9)	4 (9.3)	1 (1.9)
	Paraesthesia	1 (1.9)	0 (0)	0 (0)
	Diarrhoea	1 (1.9)	2 (4.7)	0 (0)
	Constipation	0 (0)	1 (2.3)	1 (1.9)
	Amnesia	0 (0)	2 (4.7)	0 (0)
	Concentration impairment	0 (0)	2 (4.7)	0 (0)
	Depression	0 (0)	2 (4.7)	1 (1.9)
Grade 2 [n (%)]	Vaginal inflammation	11 (21.2)	4 (9.3)	4 (7.7)
	Vaginal pain	5 (9.6)	3/43	0 (0)
	Vaginal discharge	0 (0)	2/43 (4.7)	0 (0)
	Vaginal haemorrhage	1 (1.9)	2/43 (4.7)	1 (1.9)
	Abdominal pain	1 (1.9)	0 (0)	0 (0)
	Headache	1 (1.9)	0 (0)	0 (0)
	Malaise	2/ (5.8)	4 (9.3)	0 (0)
	Fever	1/ (1.9)	0 (0)	0 (0)
	Vaginal dryness	1 (1.9)	0 (0)	0 (0)
	LUTS	3 (5.8)	3 (7.0)	4 (7.7)
	Flu-like symptoms	1 (1.9)	0 (0)	0 (0)
	Anxiety	1 (1.9)	2 (4.7)	0 (0)
	Arthralgia	0 (0)	2 (4.7)	0 (0)
	Concentration impairment	0 (0)	1 (2.3)	0 (0)
Grade 3 [n (%)]	Vaginal inflammation (including severe oedema and ulceration)	3 (5.8)	0 (0)	0 (0)
	Vaginal pain	2/ (5.8)	0 (0)	0 (0)
	Vaginal haemorrhage	0 (0)	0 (0)	0 (0)
	Abdominal pain	1/ (1.9)	0 (0)	0 (0)
	Headache	1 (1.9)	0 (0)	0 (0)
	Vaginal dryness	1 (1.9)	0 (0)	0 (0)

	Flu-like symptoms	1/ (1.9)	0 (0)	0 (0)
Other [*n* (%)]	Sexual dysfunction	17 (32.7)	12 (27.9)	10 (19.2)
	Limited in work, hobbies, family and activities	1 (1.9)	5 (11.6)	0 (0)
	Poor general health	1 (1.9)	4 (9.3)	0 (0)
	Low quality of life	0 (0)	5 (11.6)	1 (1.9)
